# Effect of flow diverter stent malposition on intracranial aneurysm hemodynamics—An experimental framework using stereoscopic particle image velocimetry

**DOI:** 10.1371/journal.pone.0264688

**Published:** 2022-03-02

**Authors:** Christoph Roloff, Philipp Berg

**Affiliations:** 1 Laboratory of Fluid Dynamics and Technical Flows, University of Magdeburg, Magdeburg, Germany; 2 Research Campus *STIMULATE*, University of Magdeburg, Magdeburg, Germany; RKH Klinikum Ludwigsburg, GERMANY

## Abstract

**Background:**

Flow-diverting stents are increasingly used for the minimally-invasive treatment of intracranial aneurysms. However, a correct positioning of such devices can be challenging due to varying vessel diameters as well as the complex anatomy of the neurovasculature. As a consequence, unsuccessful treatment outcomes are increasingly reported requiring an improvement of the understanding of stent-induced flow modification.

**Methods:**

To evaluate the effect of different degrees of flow diverter stent malposition on intra-aneurysmal hemodynamic changes, a controlled hemodynamic configuration was created using an idealized intracranial aneurysms model. Afterwards, four different treatment scenarios were reproduced comprising of 1) the ideal treatment, 2) an insufficient wall apposition in the region of the ostium, 3) a distorted device migrating into the aneurysm sac and 4) an inaccurately deployed stent due to wrong release location. For the assessment of the individual flow modifications, high-resolution stereoscopic particle image velocimetry (PIV) measurements were carried out.

**Results:**

The analysis of the precise *in-vitro* PIV measurements reveals that in all cases a considerable reduction of the cycle-averaged and peak-systolic velocity was obtained. Compared to the untreated aneurysm configuration, the flow reduction ranged from 63% (scenario 4) up to 89% (scenario 3). The ideal treatment reached a reduction of 78%, which is known to be sufficient for a successful therapy. However, inaccurate device positioning leads to increased oscillating flow towards the lateral directions reducing the chances of sufficient thrombus formation.

**Conclusions:**

High-resolution *in-vitro* PIV measurements enable an accurate quantification of the treatment efficacy for flow-diverting devices. Furthermore, insufficient treatment outcomes can be reproduces allowing for an assessment of intra-aneurysmal hemodynamic changes.

## 1. Introduction

Intracranial aneurysms (IAs) are permanent dilatations of the cerebral vessel wall and rupture can lead to severe consequences such as sudden death or irreversible disabilities. For many decades, the most effective form of treatment was open surgery, in which a metal clip was placed at the neck of the aneurysm separating it from the permanent blood flow circulation [1, 2]. However, due to interventional risks as well as long recovery periods, minimally-invasive treatment techniques were invented. In this regard, platinum coils were introduced into the aneurysm sac using a microcatheter [3–5]. Hence, the blood flow can be reduced, and a thrombosis occludes the IA naturally. Unfortunately, the placement of coils can lead to insufficient coverage of complexly shaped aneurysms or even inter-procedural perforation of thin aneurysm walls. Therefore, flow-diverting (FD) devices were developed, which are densely-braided stents that also dampen the flow into the aneurysm. However, in contrast to the coiling procedure, FDs are deployed within the parent artery and therefore treat the entire diseased vessel section and not only the symptom of this disease.

Over the past years, many clinical studies were carried out that reported the treatment success and usability of different FD devices [6–11]. On the other hand, several undesired treatment outcomes were observed, which raised concerns regarding the efficacy especially for complex aneurysms [12–18]. Kulcsár et al. [14] reported that FDs can lead to a strong reduction of the intra-saccular flow, however delayed rupture is still possible when the aneurysm is not suited for this type of therapy. Furthermore, an investigation, which includes the measurement of intra-aneurysmal pressure before, during and after FD deployment, revealed that although the flow is reduced due to the therapy, the pressure load remains almost identical [16]. Recently, Sindeev et al. [17] highlighted the importance of complete FD expansion and correct wall apposition in order to successfully treat IAs. In their case study, an incomplete deployment was associated with the occurrence of intimal hyperplasia. Overall, a literature review by Rouchaud et al. [15] demonstrates that many giant aneurysms treated with FDs tend to experience delayed rupture (almost 50%). Additionally, treatment complication rates of 18% overall and up to 27% in the posterior circulation were reported [8].

To address the described clinical observations, this experimental study focusses on a detailed investigation of undesired, but potentially occurring treatment outcomes. Therefore, a framework with controlled conditions was established using an idealized IA as well as four different deployment scenarios. Within this framework the effect of potential FD treatments (containing variants of wall apposition) was evaluated using highly-resolved *in-vitro* measurements based on stereoscopic particle image velocimetry (PIV). In this regard, an experimental environment was established to provide precise flow information for future comparisons and evaluate the effect of different degrees of FD stent malposition on intra-aneurysmal hemodynamic changes. Furthermore, the flow-related effects of undesired deployment results can be predicted enabling physicians to decide whether treatment adjustment is needed or not.

## 2. Materials and methods

### 2.1 Case description

**Fig 1:**
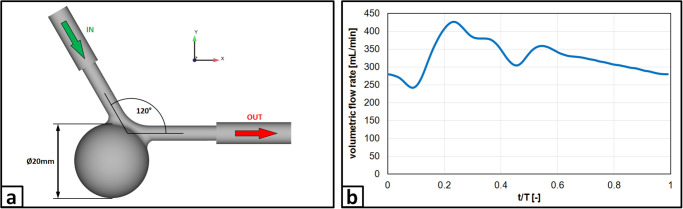
Investigated aneurysm case. a) Sketch of the idealized aneurysm geometry representing a typical candidate of a saccular malformation occurring in the neurovasculature; b) Phase-averaged flow rate as measured by the ultrasound flow meter.

For this study, an idealized spherical side wall aneurysm with a diameter of 20 mm was chosen, which represents a common aneurysm size for endovascular treatment. The parent vessel featured a diameter of 4 mm (representative diameter for a cerebral vessel such as the middle cerebral artery) and was bent to an angle of 120° (see sketch presented in panel a) of Fig 1). This controlled situation enables the assessment of device-related flow modifications and hence is suitable for the desired FD efficacy quantification.

From the 3D CAD model, a transparent phantom model for the PIV measurements was manufactured using a lost core technique resulting in a silicone block incorporating the hollow vessel structure. The refractive index of the silicone was measured to be n_silicone_ = 1.4113 at 22°C (Abbemat 200, Anton Paar, Ostfildern, Germany). For the blood analogue liquid (BAL), a mixture of distilled water, glycerin, sodium iodide and sodium thiosulphate was used, which matched the refractive index of the silicone block (n_BAL_ = 1.4109) as well as relevant fluid dynamic properties compared to physiologic arterial flow, i.e. its density of ρ_BAL_ = 1221 kg⋅m^-3^ and dynamic viscosity of μ_BAL_ = 5∙10–3 Pa⋅s result in a kinematic viscosity of ν_BAL_ = 4.1∙10–6 m^2^⋅s^-1^. This corresponds to a dynamic viscosity of real blood of μ_blood_ = 3.9∙10–3 Pa⋅s (assuming a density of ρ_blood_ = 1060 kg⋅m^-3^). Small resin microspheres doped with Rhodamine B (diameter d = 10.46 ± 0.18 μm, density ρ = 1510 kg⋅m^-3^) were used as seeding for the PIV measurements.

### 2.2 Flow diverter treatment scenarios

**Fig 2:**
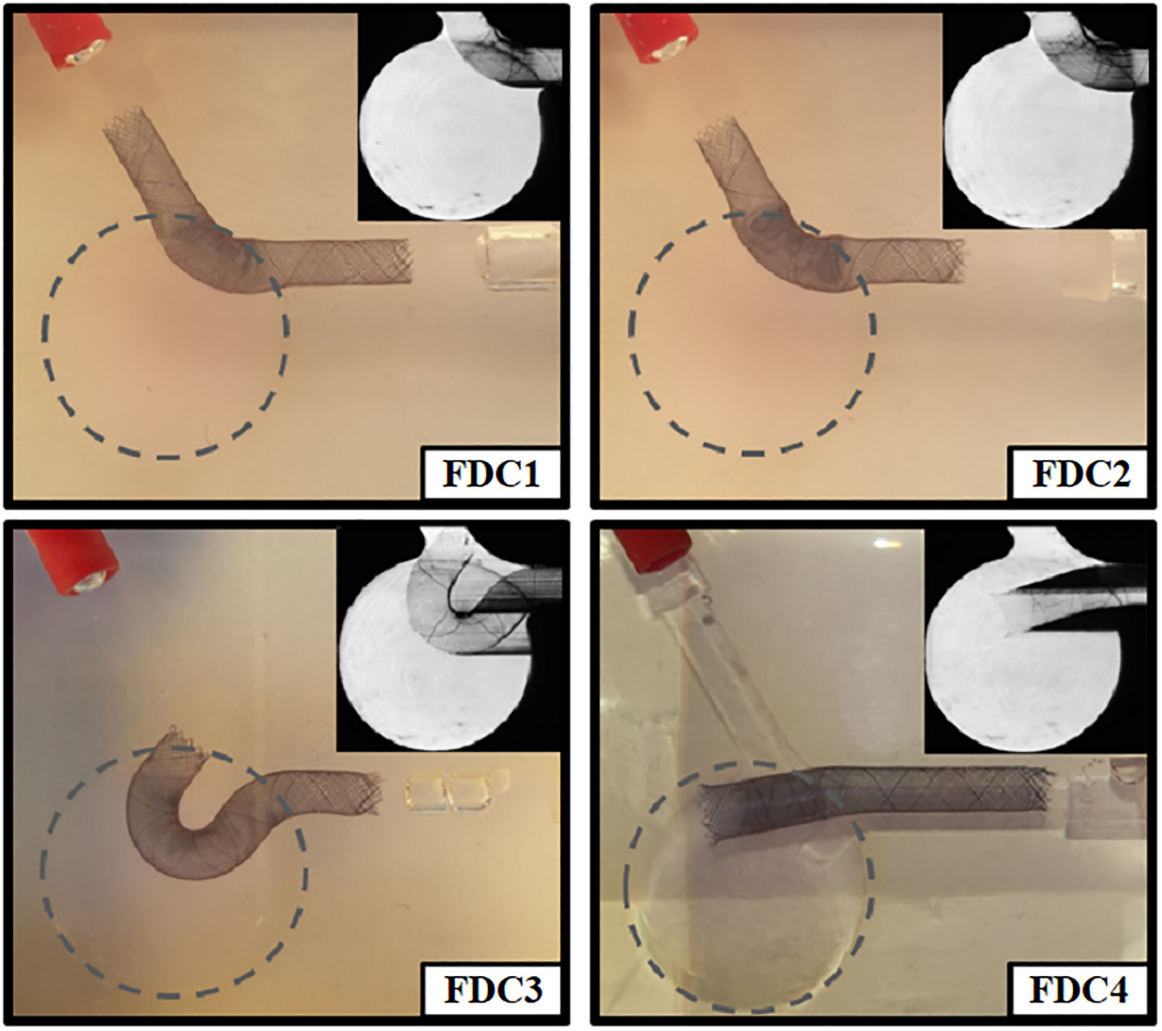
Illustration of the investigated treatment scenarios with Flow Diverter Cases 1 to 4 (FDC1 to FDC4). The real deployment within the silicone phantom model is shown (FDC1-FDC3: phantom mostly filled with index matching fluid, FDC4: phantom filled with water leading to slight refractions) as well as the PIV measurement plane containing a detailed view of the flow diverter location resulting from long exposure flow tracer reconstructions (upper right corner).

To obtain a reference solution, measurements were carried out in the empty silicone model (Case “no FD”). However, for the various treatment scenarios an Acandis Derivo 4,5 x 30 embolization device (Acandis GmbH, Pforzheim, Germany) was deployed in the parent vessel of the phantom at four different positions–**Case “FDC1”**: The device was deployed in a close-fitting, symmetrical manner with respect to the parent vessel. This configuration represents the ideal clinical treatment outcome with perfect wall apposition; **Case “FDC2”**: The FD device was dragged softly inside the aneurysm resulting in a strut compression in the region of the ostium, an insufficient wall apposition and a reduced longitudinal extension in both sides of the parent vessel. In reality, this can occur especially in vessel sections with strong curvature; **Case “FDC3”**: The axial center of the FD was drastically dragged into the aneurysm leading to a curved part of the device extending through the neck into the sac with one end just connecting to the inlet vessel. This configuration simulates a scenario, when the FD was loosely released and migrates into the aneurysm sac due to the pulsatile nature of blood flow; **Case “FDC4”**: The FD was moved downstream into the outlet vessel such that one end of the device was freely suspended in the aneurysm sac. This case represents the situation, when the deployment started at a very distal location or an under-sizing of the device occurred. Consequently, the proximal part of the stent is not sufficiently located in the vessel section and remains or regresses into the aneurysm sac. A visual representation of all four treatment scenarios as well as a detailed view resulting from long exposure PIV reconstructions is given in Fig 2.

### 2.3 In-vitro investigation

**Fig 3:**
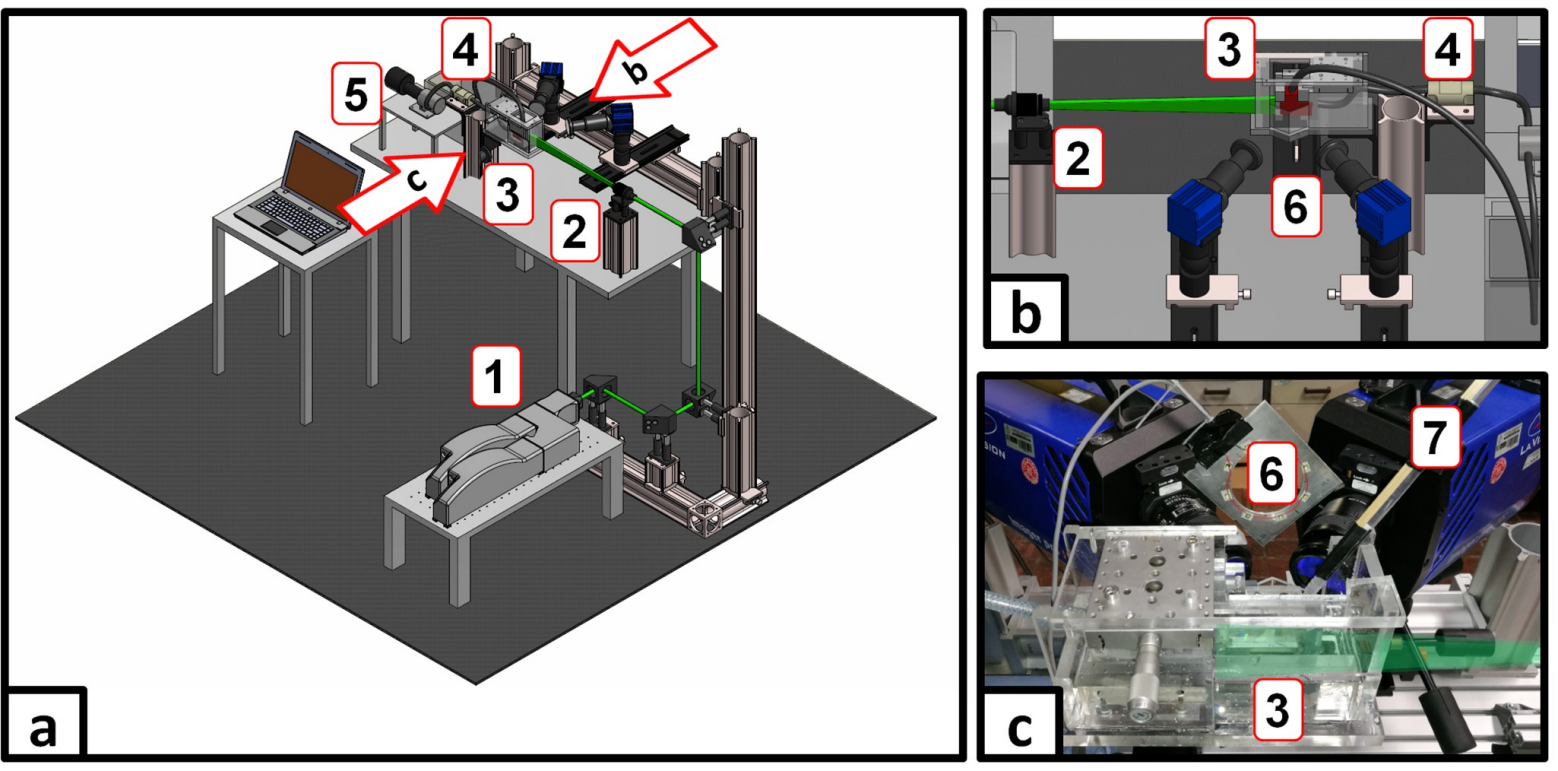
Experimental setup. (a) Principle sketch of the stereoscopic PIV setup involving laser (1), sheet optics (2), acrylic glass box with the phantom (3), flow meter (4) and gear pump (5); (b) Detailed view of (a) as seen from behind with the stereoscopic PIV camera pair (6); (c) Photography of the real measurement setup including the straight inlet tubing (7) as well as the estimated propagation of the laser light sheet.

During the PIV measurements, the phantom was placed inside a transparent acrylic box with two inclined walls and filled with index matching fluid. A laser light sheet was directed to illuminate the sagittal plane of the aneurysm. The two stereoscopic PIV highspeed cameras (sCMOS, 2560×2160 pixel) observed the flow through the inclined windows from the side of the acrylic box to minimize optical aberrations such as astigmatism. A micro-gear pump (HNP Mikrosysteme, Schwerin, Germany) delivered the periodic flow (see panel b) of Fig 1), which was monitored by an ultrasonic flow meter (Sonotec, Halle, Germany). It results in a maximum Reynolds number of Re = 1025 and a Womersley number of Wo = 2.54. To ensure a fully developed laminar flow profile, the inlet connector to the phantom consisted of a 500 mm straight tubing. The PIV double frame recordings were conducted at a frequency of 0.5 kHz (triggered by the pump control), where the interframe time was set between 200 μs (noFD) and 900 μs (FDC3). For each scenario, 36 periodic cycles were recorded resulting in 36.000 double frame pairs accordingly. Velocity processing was conducted via DaVis 8.4.0 (LaVision, Göttingen, Germany) using a multi-pass stereo cross-correlation with a final interrogation window size of 32×32 px and 50% overlap resulting in one velocity vector every 141 μm. The velocity fields were then phase-averaged for further analysis. A schematic sketch of the measurement setup together with an image of the actual measurement equipment is shown in Fig 3.

### 2.4 Qualitative and quantitative analysis

For the assessment of each FD efficacy, qualitative analysis was carried out based on the results obtained in the five *in-vitro* experiments. Here, the sagittal plane of the symmetric aneurysm was chosen since it reveals the regions of highest device impact. Furthermore, regions of high and low flow can be identified, which can be crucial for the evaluation of wall shear stresses and shear rates to potentially address IA rupture risk and thrombus formation, respectively.

Afterwards, quantitative comparisons were performed focusing on the cycle-averaged velocity (U_mean_) and the standard deviation of the out-of-plane velocity component (U_z_STD_), respectively. Furthermore, the velocity curves at three characteristic locations were monitored for each configuration (inflow jet, aneurysm dome, proximal area of the ostium) and finally, the disturbance of the flow fields was assessed using the oscillatory velocity index (OVI, see Eq 1, while *v*_*i*_ is the instantaneous flow velocity vector and *T* is the duration of one cardiac cycle) [19].


$$OVI=\frac{1}{2}\left(1-\frac{\left|\int_{0}^{T}v_{i}dt\right|}{\int_{0}^{T}\left|v_{i}\right|dt}\right)$$ (1)

## 3. Results

**Fig 4:**
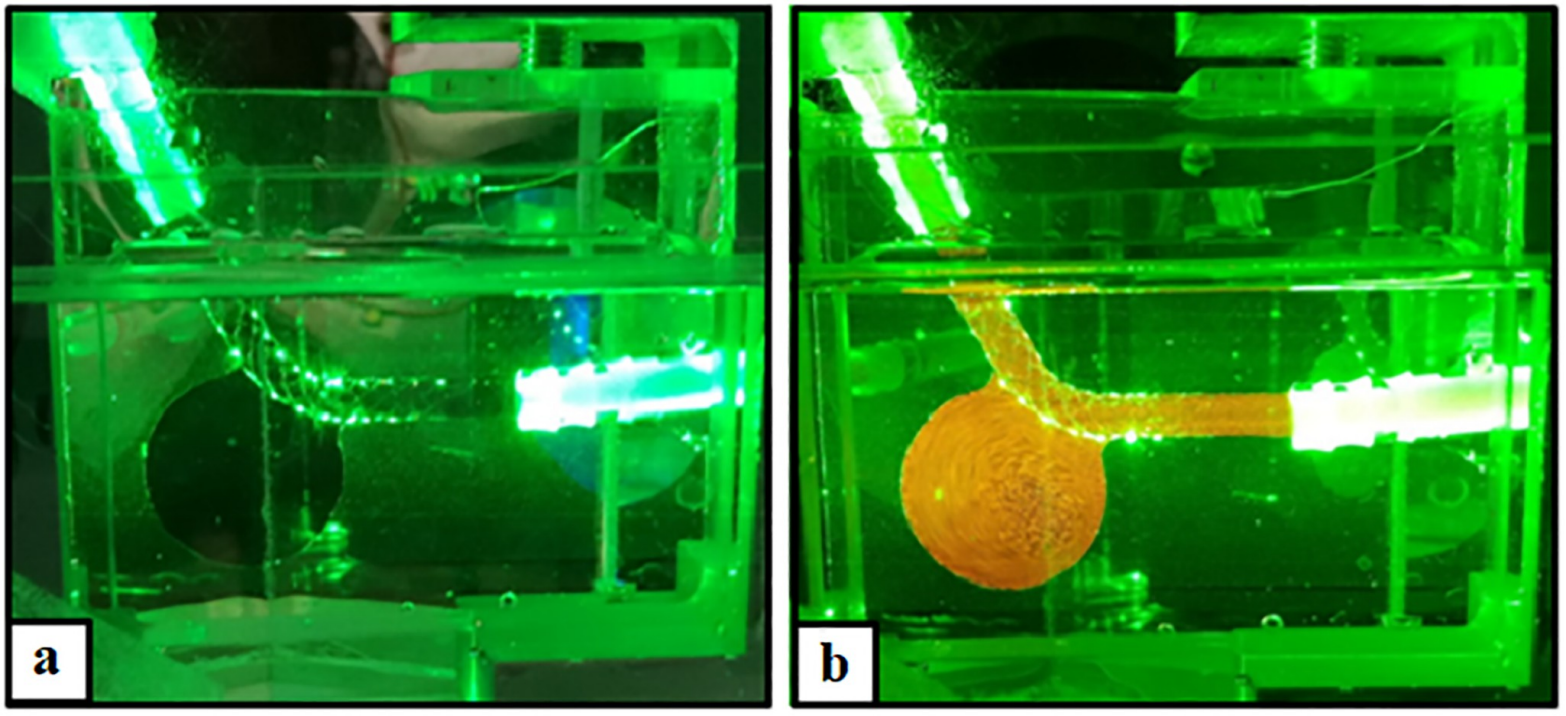
Flow measurements with and without tracer particles. Illustration of the illuminated phantom model containing a representative endovascular device (left); Addition of PIV fluorescent tracer particles revealing the vortical flow structure within the intracranial aneurysm (right).

The complex stereoscopic PIV setup described in section 2.3 was successfully established. As shown in Fig 4, flow measurements with and without tracer particles are feasible for the treated IA model demonstrating the high spatial resolution of the underlying methodology.

In the following, the results of the experimental investigation of different (undesired) FD treatments are presented. First, qualitative comparisons are provided to assess the occurring flow structures and enable a visual evaluation of each time-point during the cardiac cycle. This allows for a visual analysis of flow phenomena, which potentially leads to increased risk of rupture or—ideally—to the promotion of a thrombosis. Afterwards, a quantitative analysis reveals the real efficacy of the FD configurations as well as existing differences between the individual *in-vitro* assessments.

### 3.1 Qualitative comparison

**Fig 5:**
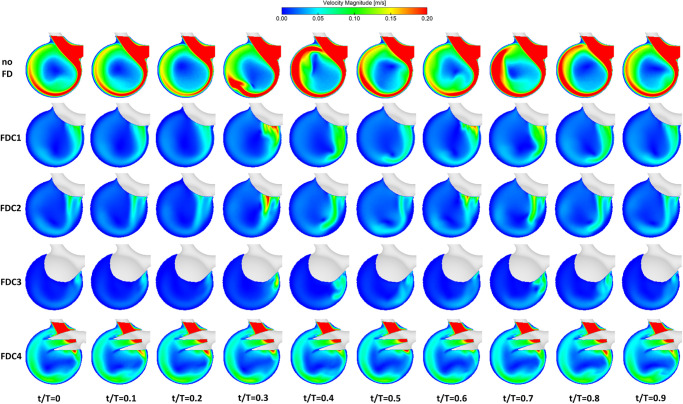
Qualitative PIV measurement results. Comparison of all treatment configurations (FDC1-4) at ten time steps during the cardiac cycle. Notice the clear differences of each FD efficacy compared to the pre-treatment scenario in the first row.

As illustrated in Fig 5, highest velocities occur in the parent vessel due to its relatively small diameter compared to the aneurysm. One can notice that the fluid enters the aneurysm at the distal part of the ostium and the corresponding inflow jet impinges on the opposite section of the aneurysm wall. Afterwards, the flow aligns clockwise near the aneurysmal lumen, until it returns into the parent vessel in distal direction. This flow structure results in the formation of a stagnation zone in the center of the aneurysm with velocity values close to zero. Due to the pulsatile nature of the inflow signal, one can notice the effect of the inflow jet, especially in the pre-treatment configuration during peak-systole. In addition to the static results presented in Fig 5, time-dependent animations of the velocity distribution can be found in the supporting material S1 File.

After deploying the FD device in the desired location (FDC1), the velocity values strongly decrease throughout the complete cardiac cycle. Since increased values only remain in the inflow jet region, the area of flow stagnation slightly shifts towards the distal part of the aneurysm. As expected, the second treatment option (FDC2), provoking an insufficient wall apposition opposite to the ostium, shows an almost identical flow-diverting effect. Here, the jet enters slightly more proximal leading to less flow alignment on the aneurysm lumen and a higher velocity close to the aneurysm center. The third configuration (FDC3) results in the strongest flow reduction due to the increased area of the perfused device. However, due to the positioning a considerable part of the malformation is covered and hence a successful long-term treatment outcome is questionable. Furthermore, side-effects for the distal vascular system are expected. Finally, the fourth treatment scenario (FDC4) representing an inappropriate estimation of the device landing zone which leads to both insufficient flow reduction and areas with increased velocity magnitude remaining within the aneurysm. This is particularly dangerous since the desired thrombosis may not set in and undesired flow-diverting effects can be induced in the distal part of the healthy vessel.

### 3.2 Quantitative comparison

To confirm the qualitative observation above, both cycle-averaged and peak-systolic velocity reductions are quantified in Table 1. It is noticeable that for each configuration the effect of the FD placement is higher at the time of largest inflow compared to the mean values. To account for flow occurring in the normal direction of the considered measurement plane, the normalized out-of-plane fluctuation was introduced. Interestingly, the value increases only by 44% and 25% for the cases FDC1 and FDC2, respectively, compared to the untreated configuration. However, for the treatment scenarios with poor wall apposition (FDC3 and FDC4) this value rises even 10-fold underlining the different impact of flow diverter topology in these cases on the resulting aneurysm flow compared to FDC1 and FDC2.

**Table 1:** Quantification of the relative velocity reduction of each FD case referring to the untreated aneurysmal flow. For all configurations both peak-systolic as well as cycle-averaged values are provided.

	U_mean_ [m/s]				
	no FD	FDC1	FDC2	FDC3	FDC4
**cyclic max**	0.153	0.031	0.026	0.017	0.047
**cyclic mean**	0.119	0.026	0.021	0.013	0.043
	U_z_STD_ / U_mean_ [-]				
**cyclic mean**	0.036	0.052	0.045	0.364	0.368

**Fig 6:**
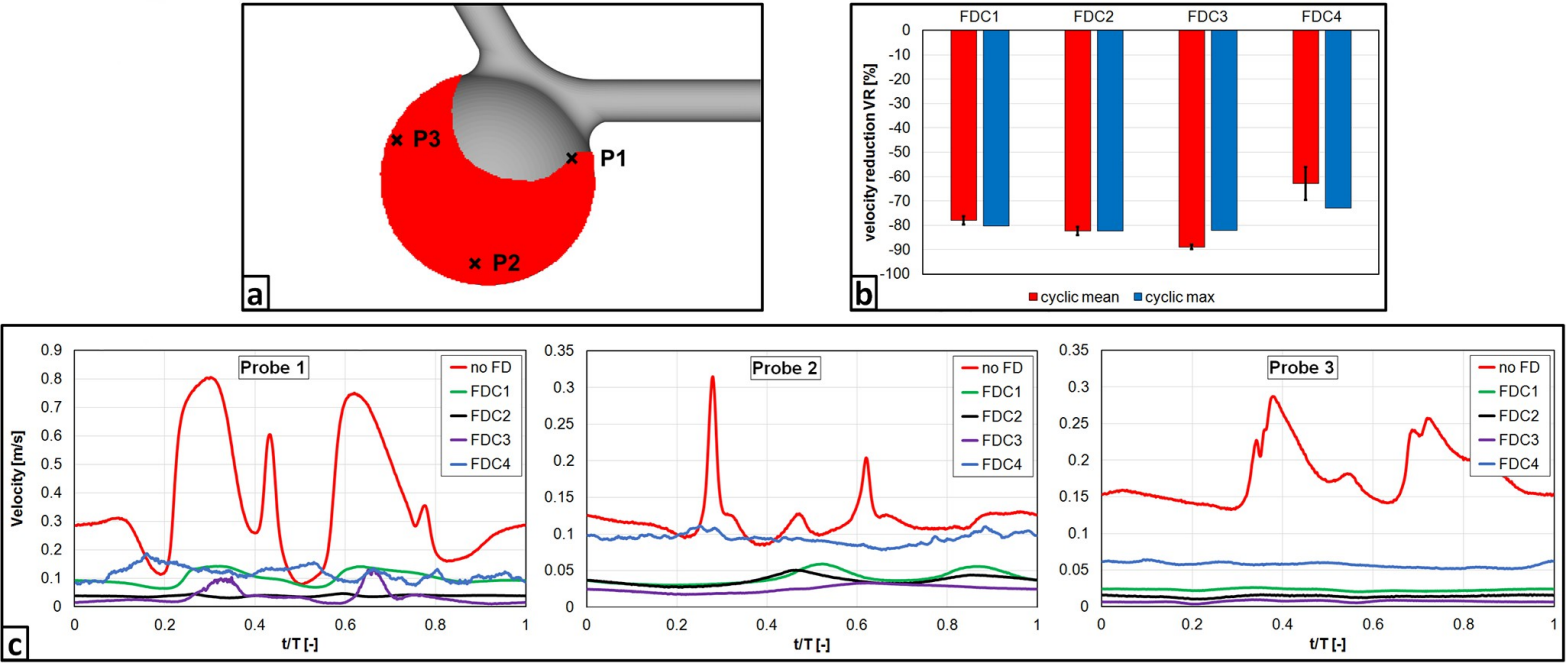
Quantitative PIV measurement results. a) Location of processed flow area (red) and velocity probes (P1 –inflow jet; P2 –aneurysm dome; P3 –close to the proximal area of the ostium); b) Quantification of the cycle-averaged and peak-systolic velocity reduction for each considered deployment scenario. Temporal velocity profiles over the averaged cardiac cycle at three characteristic probe locations in the aneurysm.

Additionally, Fig 6 illustrates the velocity reduction for the different treatment scenarios (FDC1-4). Compared to the untreated aneurysm configuration, the flow was reduced ranging from 63% (FDC4) up to 89% (FDC3). The ideal treatment (FDC1) reached a reduction of 78%, which is known to be sufficient for a successful therapy.

Furthermore, velocity curves throughout one cardiac cycle were monitored at three representative locations within the aneurysm. First, the high velocity values occurring in the inflow jet can be drastically reduced by all flow-diverter configurations and range at an almost identical level (see Fig 6, probe 1). For the aneurysm dome, which represents the second probe location, a similar behavior can be noticed with the exception of FDC4. Here, the velocity is nearly the same compared to the pre-interventional state, however, the systolic velocity peaks are damped. Finally, the third probe, located close to the proximal part of the ostium, reveals a clear reduction of the post-treatment velocity with very low values except for FDC4. This is caused by the inappropriate placement of the device resulting in a less effective flow reduction (recall Fig 2).

**Fig 7:**
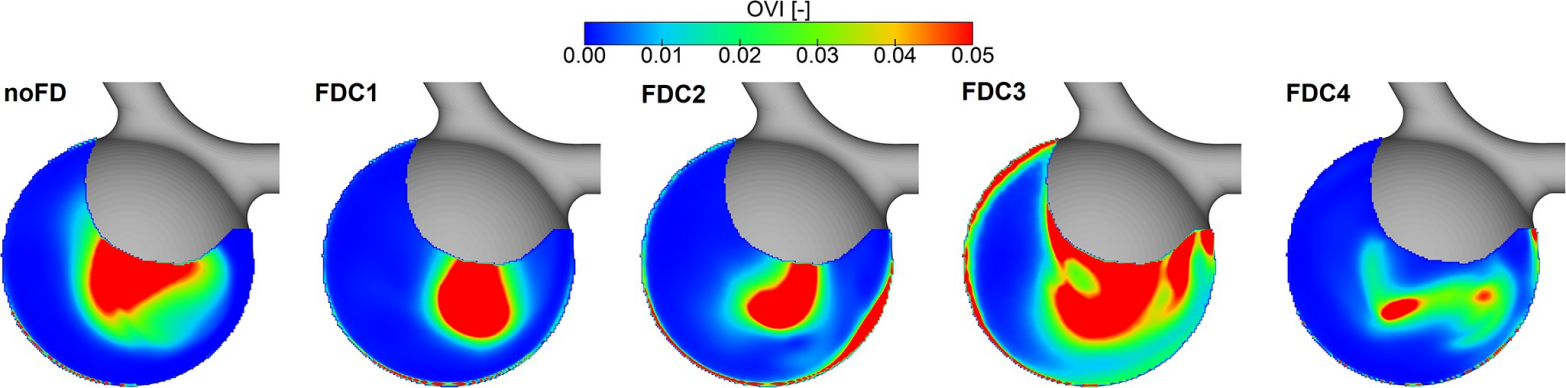
Illustration of the oscillatory velocity index (OVI) to account for device-induced disturbance of the time-dependent flow fields. From left to right: no FD, FDC1, FDC2, FDC3, FDC4.

To further assess the influence that is caused by different (potentially unsuccessful) FD treatments, the disturbance of the flow fields was quantified. Here, the OVI represents the temporal changes of the velocity vector over one cardiac cycle (see Fig 7). It can be noticed that the region of increased OVI occurs in the center of the aneurysm for the successful treatment (FDC1). The slightly insufficient wall apposition (FDC2) leads to increased OVI values in the flow jet impingement zone, which can represent an area of potentially higher rupture risk. For the third configuration (FDC3) an even stronger increase of OVI is visible throughout the whole aneurysm sac indicating the need to avoid such a treatment outcome. A potential reason for the high oscillations in z-direction is a lateral flow around the FD. Finally, although the flow reduction was not as effective as for the other cases, the oscillations within the aneurysm treated with FDC4 are smaller compared to the previous cases. This might be due to the free end of the FD damping the kinetic energy and leading to smoothed flow curves throughout the cardiac cycle (recall Fig 6).

## 4. Discussion

Minimally-invasive treatment of IAs is increasingly used compared to traditional techniques such as surgical clipping [20]. Endovascular FD placement, in particular, is the method of choice for specific aneurysm configurations since the device is placed below the aneurysm ostium avoiding the risk of perforating thin vessel walls (e.g., due to insufficient coiling). Furthermore, the full diseased vessel section, which caused the formation of these permanent dilations, is stabilized leading to the formation of a novel endothelial layer [21, 22].

Unfortunately, the treatment risk still remains higher compared to the natural rupture risk of intracranial aneurysms [23, 24]. Procedural complications can result in intimal hyperplasia, perforator infarction, intraparenchymal hemorrhage or even procedure-related morbidity and mortality [25, 26]. In a systematic review and meta-analysis Cagnazzo et al. found that flow-diversion treatment of ruptured intracranial aneurysms is associated with a complication rate of 18% and even 27% for ruptured posterior circulation aneurysms [8].

Therefore, improvements regarding the treatment outcome are crucial, which requires a better understanding of the underlying flow phenomena. In this regard, Anderson et al. used phase-contrast MRI to measure flow reduction due to FD placement [27]. They found that the flow velocity was significantly decreased, and intra-aneurysmal flow patterns were maintained. Another pilot study comparing successful and unsuccessful treatments of aneurysms occurring at the internal carotid artery demonstrated the need to obtain a flow reduction below a critical absolute inflow value instead of simply a high relative reduction [28]. Recently, Chodzyǹski et al. suggest that flow complexity should be considered when selecting a FD since increased pulsatility altered the performance of the device [29].

To investigate the effect of various treatment scenarios with poor wall apposition, this study created a well-defined observational environment. Specifically, a silicone phantom model of an idealized IA was manufactured and treated with one optimal and three undesirable FD configurations. Afterwards, precise time-dependent *in-vitro* measurements were carried out using high-resolution stereoscopic PIV enabling the assessment of occurring flow structures as well as the specific treatment efficacy. Although real IAs present with a highly patient-specific size and shape, this artificial environment avoids the occurrence of individual hemodynamic effects. Nevertheless, the presented framework can be applied to any arbitrary malformation and treatment configuration in the future.

The results of this study reveal that the successful treatment scenario (FDC1) led to a strong reduction of the spatially averaged intra-saccular velocity at peak systole and for the averaged cardiac cycle. Furthermore, the flow structure involving a smooth alignment of the stable vortex near the wall was maintained, which ensures no undesired side-wall effects. A slight migration of the flow diverter through the aneurysm ostium (FDC2) resulted in a minor increase of the velocity reduction. However, the incomplete wall apposition on the opposite side of the parent vessel impedes the formation of a novel endothelial layer, which is known to potentially cause long-term complications [30, 31]. The third treatment scenario (FDC3) nominally led to the strongest reduction of the blood flow due to the doubled diverting effect. Hence, it theoretically appears to be the best therapy outcome, however, even if the reduction is beneficial for the initiation of a thrombus, the risk of a complete occlusion of the vessel remains (due to endothelialization and in-stent thrombosis) [32]. Finally, the fourth configuration (FDC4), which could occur in practice due to an inaccurate choice of the stent release, shows a lower velocity reduction effect compared to the other scenarios. Furthermore, additional long-term complications can appear, if an unjustified under- or oversizing for the stent was selected [33]. Therefore, it is of highest importance to appropriately choose a FD that fits the corresponding parent vessel size and shape as well as takes into account potentially-covered side branches in the vicinity of the aneurysm.

Apart from the treatment comparisons, an enormous amount of high-resolution measurement data was acquired within this study, which can serve as a reference for future validations, e.g., as benchmarks for in-house developed fluid flow solvers and can be arbitrarily extended depending on the particular interest [34, 35]. The corresponding time-dependent datasets for all considered configurations were made publicly available in the supporting material S1 Dataset. Additional to these *in-vitro* PIV measurements, *in-silico* investigations based on computational fluid dynamics (CFD) are feasible to enable an easier variation for parameters of interest (e.g., vessel sizes, aneurysm shapes, FD designs). Initial hemodynamic simulations were carried out to obtain a more detailed insight especially in the vicinity of small stent struts. Here, an advanced explicit fast virtual stenting approach was applied allowing for the consideration of arbitrary treatment scenarios [36, 37]. In the supporting material S2 File, a video of these simulations is provided.

In the near future, it can be expected that the treatment of IAs becomes more and more individualized. This includes the development of a variety of FD variants specialized for different locations and types of aneurysms [38]. The framework presented within this study provides a valuable opportunity to assess the corresponding FD efficacy as well as the effect of different degrees of FD stent malposition on intra-aneurysmal hemodynamic changes. Additionally, advanced measurement techniques such as x-ray PIV can be applied to gain further insight into the underlying flow phenomena [39].

### Limitations

This study has several limitations which need to be mentioned. First, only one IA as well as one FD stent was used. Hence, the results cannot be generalized for all types of aneurysms or treatment options (e.g., coils or WEB-devices), but the rational was to create a controlled environment, and the experiments for the chosen configurations were already highly time-consuming and expensive. Furthermore, the configurations FDC3 and FDC4 might not occur in clinical practice, but this study demonstrates the feasibility of assessing arbitrary (undesired) treatment configurations. Although a reproduction of the stent deployment scenarios might be difficult, the measured data are openly accessible and can be used for further comparisons.

Second, to enable an evaluation of the real device efficacy, *in-vivo* measurements, e.g., using phase-contrast MRI could be used [40, 41]. This would overcome the limitation that interfering structures such as bones cannot be assessed with optical PIV. However, due to the limited spatial and temporal MR-resolution as well as imaging artifacts due to movements and device distortions, a precise quantification is not feasible. Therefore, the *in-vitro* approach used in this study represents the state-of-the-art. Nevertheless, the effect of the pump pulse shape as well as potential wall compliance of the aneurysm model should be carefully considered in this regard [42].

Third, the treatment scenarios created here are hardly reproducible in reality since each aneurysm completely differs in size and shape and the adjacent parent vessel can present with strong curvature changes. However, the motivation of this study was to initially quantify the effect of undesired treatment outcomes on the underlying hemodynamics and provide high-quality measurement data for future investigations, which should include complex vessel anatomies containing torsional shapes and varying proximal and distal angles.

## 5. Conclusions

This study evaluates the effect of various FD treatment scenarios (possessing variants of good and poor wall apposition) for IAs using stereoscopic PIV. Through this, high-resolution measurement data were acquired, which were made freely available and can be used for external comparisons and validation purposes. The results of this study suggest that incorrect stent deployment can lead to sufficient intra-aneurysmal flow reduction, but potential complications due to a disturbed flow behavior must be evaluated in the future using a realistic neurovasculature. Furthermore, the presented framework consisting of phantom manufacturing, FD deployment and precise flow acquisition enables the quantification of arbitrary treatment outcomes and can be applied to complex aneurysm shapes and novel devices in the future. Hence, undesired flow-related effects are assessable supporting neurointerventionalists during therapy planning as well as education.

## Supporting information

S1 File Animation of the time-dependent velocity contours acquired using stereoscopic PIV. (AVI)

S2 File Animation of the time-dependent velocity contours numerically acquired using computational fluid dynamics (CFD). (AVI)

S1 Dataset Time-dependent velocity fields acquired using stereoscopic PIV and processed into EnSight-format. (ZIP)

S2 Dataset Time-dependent velocity fields acquired using stereoscopic PIV and processed into EnSight-format. (RAR)

S3 Dataset Time-dependent velocity fields acquired using stereoscopic PIV and processed into EnSight-format. (RAR)

S4 Dataset Time-dependent velocity fields acquired using stereoscopic PIV and processed into EnSight-format. (RAR)

S5 Dataset Time-dependent velocity fields acquired using stereoscopic PIV and processed into EnSight-format. (RAR)

## References

[1] Britz GW, Salem L, Newell DW, Eskridge J, Flum DR. Impact of surgical clipping on survival in unruptured and ruptured cerebral aneurysms: a population-based study. Stroke 2004;35(6):1399–403. doi: 10.1161/01.STR.0000128706.41021.01 15118171

[2] Bruneau M, Amin-Hanjani S, Koroknay-Pal P, Bijlenga P, Jahromi BR, Lehto H, et al. Surgical Clipping of Very Small Unruptured Intracranial Aneurysms: A Multicenter International Study. Neurosurgery 2016;78(1):47–52. doi: 10.1227/NEU.0000000000000991 26317673

[3] Molyneux AJ, Kerr RSC, Yu L-M, Clarke M, Sneade M, Yarnold JA, et al. International subarachnoid aneurysm trial (ISAT) of neurosurgical clipping versus endovascular coiling in 2143 patients with ruptured intracranial aneurysms: a randomised comparison of effects on survival, dependency, seizures, rebleeding, subgroups, and aneurysm occlusion. Lancet 2005;366(9488):809–17. doi: 10.1016/S0140-6736(05)67214-5 16139655

[4] Brinjikji W, Kallmes DF, Kadirvel R. Mechanisms of Healing in Coiled Intracranial Aneurysms: A Review of the Literature. AJNR Am J Neuroradiol 2015;36(7):1216–22. doi: 10.3174/ajnr.A4175 25430855 PMC4939243

[5] Zhao B, Yin R, Lanzino G, Kallmes DF, Cloft HJ, Brinjikji W. Endovascular Coiling of Wide-Neck and Wide-Neck Bifurcation Aneurysms: A Systematic Review and Meta-Analysis. AJNR Am J Neuroradiol 2016;37(9):1700–5. doi: 10.3174/ajnr.A4834 27256850 PMC7984700

[6] D’Urso PI, Lanzino G, Cloft HJ, Kallmes DF. Flow diversion for intracranial aneurysms: a review. Stroke 2011;42(8):2363–8. doi: 10.1161/STROKEAHA.111.620328 21737793

[7] Briganti F, Leone G, Marseglia M, Mariniello G, Caranci F, Brunetti A, et al. Endovascular treatment of cerebral aneurysms using flow-diverter devices: A systematic review. Neuroradiol J 2015;28(4):365–75. doi: 10.1177/1971400915602803 26314872 PMC4757311

[8] Cagnazzo F, Di Carlo DT, Cappucci M, Lefevre P-H, Costalat V, Perrini P. Acutely Ruptured Intracranial Aneurysms Treated with Flow-Diverter Stents: A Systematic Review and Meta-Analysis. AJNR Am J Neuroradiol 2018;39(9):1669–75. doi: 10.3174/ajnr.A5730 30049721 PMC7655299

[9] Chalouhi N, Tjoumakaris S, Starke RM, Gonzalez LF, Randazzo C, Hasan D, et al. Comparison of flow diversion and coiling in large unruptured intracranial saccular aneurysms. Stroke 2013;44(8):2150–4. doi: 10.1161/STROKEAHA.113.001785 23723311

[10] Silva MA, See AP, Khandelwal P, Mahapatra A, Frerichs KU, Du R, et al. Comparison of flow diversion with clipping and coiling for the treatment of paraclinoid aneurysms in 115 patients. J Neurosurg 2018:1–8. doi: 10.3171/2018.1.JNS171774 29932380

[11] Zhang M, Li Y, Sugiyama S-I, Verrelli DI, Matsumoto Y, Tominaga T, et al. Incomplete stent expansion in flow-diversion treatment affects aneurysmal haemodynamics a quantitative comparison of treatments affected by different severities of malapposition occurring in different segments of the parent artery. Int J Numer Method Biomed Eng 2021:e3465. doi: 10.1002/cnm.3465 33847467

[12] Cohen JE, Gomori JM, Moscovici S, Leker RR, Itshayek E. Delayed complications after flow-diverter stenting: reactive in-stent stenosis and creeping stents. J Clin Neurosci 2014;21(7):1116–22. doi: 10.1016/j.jocn.2013.11.010 24524952

[13] Guédon A, Clarençon F, Di Maria F, Rosso C, Biondi A, Gabrieli J, et al. Very late ischemic complications in flow-diverter stents: a retrospective analysis of a single-center series. J Neurosurg 2016;125(4):929–35. doi: 10.3171/2015.10.JNS15703 26824382

[14] Kulcsár Z, Houdart E, Bonafé A, Parker G, Millar J, Goddard AJP, et al. Intra-aneurysmal thrombosis as a possible cause of delayed aneurysm rupture after flow-diversion treatment. AJNR Am J Neuroradiol 2011;32(1):20–5. doi: 10.3174/ajnr.A2370 21071538 PMC7964960

[15] Rouchaud A, Brinjikji W, Lanzino G, Cloft HJ, Kadirvel R, Kallmes DF. Delayed hemorrhagic complications after flow diversion for intracranial aneurysms: a literature overview. Neuroradiology 2016;58(2):171–7. doi: 10.1007/s00234-015-1615-4 26553302 PMC4849277

[16] Schneiders JJ, VanBavel E, Majoie CB, Ferns SP, van den Berg R. A flow-diverting stent is not a pressure-diverting stent. AJNR Am J Neuroradiol 2013;34(1):4. doi: 10.3174/ajnr.A2613 21852372 PMC7966342

[17] Sindeev S, Prothmann S, Frolov S, Zimmer C, Liepsch D, Berg P, et al. Intimal hyperplasia after aneurysm treatment by flow diversion: A case report. World Neurosurgery 2018. doi: 10.1016/j.wneu.2018.10.107 31108073

[18] Zhou G, Su M, Yin Y-L, Li M-H. Complications associated with the use of flow-diverting devices for cerebral aneurysms: a systematic review and meta-analysis. Neurosurg Focus 2017;42(6):E17. doi: 10.3171/2017.3.FOCUS16450 28565981

[19] Tanioka S, Ishida F, Kishimoto T, Tsuji M, Tanaka K, Shimosaka S, et al. Quantification of hemodynamic irregularity using oscillatory velocity index in the associations with the rupture status of cerebral aneurysms. J Neurointerv Surg 2019;11(6):614–7. doi: 10.1136/neurintsurg-2018-014489 30670624

[20] Seibert B, Tummala RP, Chow R, Faridar A, Mousavi SA, Divani AA. Intracranial aneurysms: review of current treatment options and outcomes. Front Neurol 2011;2:45. doi: 10.3389/fneur.2011.00045 21779274 PMC3134887

[21] Ravindran K, Salem MM, Alturki AY, Thomas AJ, Ogilvy CS, Moore JM. Endothelialization following Flow Diversion for Intracranial Aneurysms: A Systematic Review. AJNR Am J Neuroradiol 2019;40(2):295–301. doi: 10.3174/ajnr.A5955 30679207 PMC7028638

[22] Ravindran K, Casabella AM, Cebral J, Brinjikji W, Kallmes DF, Kadirvel R. Mechanism of Action and Biology of Flow Diverters in the Treatment of Intracranial Aneurysms. Neurosurgery 2020;86(Suppl 1):S13–S19. doi: 10.1093/neuros/nyz324 31838528 PMC6911734

[23] Bonney PA, Connor M, Fujii T, Singh P, Koch MJ, Stapleton CJ, et al. Failure of Flow Diverter Therapy: Predictors and Management Strategies. Neurosurgery 2020;86(Suppl 1):S64–S73. doi: 10.1093/neuros/nyz305 31838530

[24] Rajah G, Narayanan S, Rangel-Castilla L. Update on flow diverters for the endovascular management of cerebral aneurysms. Neurosurg Focus 2017;42(6):E2. doi: 10.3171/2017.3.FOCUS16427 28565980

[25] Brinjikji W, Murad MH, Lanzino G, Cloft HJ, Kallmes DF. Endovascular treatment of intracranial aneurysms with flow diverters: a meta-analysis. Stroke 2013;44(2):442–7. doi: 10.1161/STROKEAHA.112.678151 23321438

[26] Brinjikji W, Lanzino G, Cloft HJ, Siddiqui AH, Kallmes DF. Risk Factors for Hemorrhagic Complications following Pipeline Embolization Device Treatment of Intracranial Aneurysms: Results from the International Retrospective Study of the Pipeline Embolization Device. AJNR Am J Neuroradiol 2015;36(12):2308–13. doi: 10.3174/ajnr.A4443 26251427 PMC7964264

[27] Anderson JR, Klucznik R, Diaz O, Zhang YJ, Britz GW, Grossman RG, et al. Quantification of velocity reduction after flow diverter placement in intracranial aneurysm: An ex vivo study with 3D printed replicas. Conf Proc IEEE Eng Med Biol Soc 2015;2015:7300–3. doi: 10.1109/EMBC.2015.7320077 26737977

[28] Berg P, Saalfeld S, Janiga G, Brina O, Cancelliere NM, Machi P, et al. Virtual stenting of intracranial aneurysms: A pilot study for the prediction of treatment success based on hemodynamic simulations. Int J Artif Organs 2018;41(11):698–705. doi: 10.1177/0391398818775521 29783867

[29] Chodzyǹski KJ, Uzureau P, Nuyens V, Rousseau A, Coussement G, Zouaoui Boudjeltia K. The impact of arterial flow complexity on flow diverter outcomes in aneurysms. Sci Rep 2020;10(1):10337. doi: 10.1038/s41598-020-67218-9 32587308 PMC7316819

[30] Rouchaud A, Ramana C, Brinjikji W, Ding Y-H, Dai D, Gunderson T, et al. Wall Apposition Is a Key Factor for Aneurysm Occlusion after Flow Diversion: A Histologic Evaluation in 41 Rabbits. AJNR Am J Neuroradiol 2016;37(11):2087–91. doi: 10.3174/ajnr.A4848 27390319 PMC5219872

[31] Gounis MJ, Ughi GJ, Marosfoi M, Lopes DK, Fiorella D, Bezerra HG, et al. Intravascular Optical Coherence Tomography for Neurointerventional Surgery. Stroke 2018:STROKEAHA118022315. doi: 10.1161/STROKEAHA.118.022315 30580737 PMC6541539

[32] Mühl-Benninghaus R, Haußmann A, Simgen A, Tomori T, Reith W, Yilmaz U. Transient in-stent stenosis: a common finding after flow diverter implantation. J Neurointerv Surg 2019;11(2):196–9. doi: 10.1136/neurintsurg-2018-013975 29970620

[33] Berg P, Iosif C, Ponsonnard S, Yardin C, Janiga G, Mounayer C. Endothelialization of over- and undersized flow-diverter stents at covered vessel side branches: An in vivo and in silico study. J Biomech 2016;49(1):4–12. doi: 10.1016/j.jbiomech.2015.10.047 26607220

[34] Li Y, Yoneyama Y, Isoda H, Terada M, Kosugi T, Kosugi T, et al. Haemodynamics in a patient-specific intracranial aneurysm according to experimental and numerical approaches: A comparison of PIV, CFD and PC-MRI. Technol Health Care 2021;29(2):253–67. doi: 10.3233/THC-202252 32568138

[35] Berg P, Roloff C, Beuing O, Voss S, Sugiyama S-I, Aristokleous N, et al. The Computational Fluid Dynamics Rupture Challenge 2013—Phase II: Variability of Hemodynamic Simulations in Two Intracranial Aneurysms. J Biomech Eng 2015;137(12):121008. doi: 10.1115/1.4031794 26473395

[36] Janiga G, Daróczy L, Berg P, Thévenin D, Skalej M, Beuing O. An automatic CFD-based flow diverter optimization principle for patient-specific intracranial aneurysms. J Biomech 2015;48(14):3846–52. doi: 10.1016/j.jbiomech.2015.09.039 26472308

[37] Berg P, Daróczy L, Janiga G. Virtual Stenting for Intracranial Aneurysms. In: Simone B, editor. Computing and visualization for intravascular imaging and computer-assisted stenting. Amsterdam, Netherlands: Academic Press; 2017. p. 371–411.

[38] Chiu AHY, Phillips TJ. Future Directions of Flow Diverter Therapy. Neurosurgery 2020;86(Suppl 1):S106–S116. doi: 10.1093/neuros/nyz343 31838531 PMC6911736

[39] Krebs JM, Shields A, Sharma A, Shepard LM, Ionita CN, Bednarek D, et al. Initial investigations of x-ray particle imaging velocimetry (X-PIV) in 3D printed phantoms using 1000 fps High-Speed Angiography (HSA). In: Gimi BS, Krol A, editors. Medical Imaging 2020: Biomedical Applications in Molecular, Structural, and Functional Imaging. Biomedical Applications in Molecular, Structural, and Functional Imaging; Feb. 15–20, 2020; Houston, United States: SPIE; 2020–2020. p. 39.

[40] Brina O, Bouillot P, Reymond P, Luthman AS, Santarosa C, Fahrat M, et al. How Flow Reduction Influences the Intracranial Aneurysm Occlusion: A Prospective 4D Phase-Contrast MRI Study. AJNR Am J Neuroradiol 2019;40(12):2117–23. doi: 10.3174/ajnr.A6312 31727755 PMC6975363

[41] Amili O, Schiavazzi D, Moen S, Jagadeesan B, van de Moortele P-F, Coletti F. Hemodynamics in a giant intracranial aneurysm characterized by in vitro 4D flow MRI. PLoS One 2018;13(1):e0188323. doi: 10.1371/journal.pone.0188323 29300738 PMC5754057

[42] Tupin S, Saqr KM, Ohta M. Effects of wall compliance on multiharmonic pulsatile flow in idealized cerebral aneurysm models: comparative PIV experiments. Exp Fluids 2020;61(7). doi: 10.1007/s00348-020-02998-4

